# Adapting Blood DNA Methylation Aging Clocks for Use in Saliva Samples With Cell-type Deconvolution

**DOI:** 10.3389/fragi.2021.697254

**Published:** 2021-07-29

**Authors:** Fedor Galkin, Kirill Kochetov, Polina Mamoshina, Alex Zhavoronkov

**Affiliations:** ^1^ Deep Longevity Limited, Hong Kong, China; ^2^ Insilico Medicine Hong Kong Limited, Hong Kong Science and Technology Park, Hong Kong, China; ^3^ The Buck Institute for Research on Aging, Novato, CA, United States

**Keywords:** aging, biogerontology, epigenetics, aging clock, deep learning, cell-type deconvolution, DNA methylation, domain adaptation

## Abstract

DeepMAge is a deep-learning DNA methylation aging clock that measures the organismal pace of aging with the information from human epigenetic profiles. In blood samples, DeepMAge can predict chronological age within a 2.8 years error margin, but in saliva samples, its performance is drastically reduced since aging clocks are restricted by the training set domain. However, saliva is an attractive fluid for genomic studies due to its availability, compared to other tissues, including blood. In this article, we display how cell type deconvolution and elastic net can be used to expand the domain of deep aging clocks to other tissues. Using our approach, DeepMAge’s error in saliva samples was reduced from 20.9 to 4.7 years with no retraining.

## 1 Introduction

Aging clocks are the centerpiece of the emerging longevity industry. They allow us to accurately quantify a hidden property of living organisms–the pace of aging.

Over the years the concept of aging clocks has been validated in numerous settings. An increased pace of aging has been shown to manifest in diseases, smoking, obesity, and other conditions that could be interpreted as reducing human longevity potential. At the same time, a slower pace of aging has been associated with lower mortality, and decreasing it is the aim of a new medical paradigm–longevity medicine (Bischof et al., 2021).

However, each aging clock comes with its own set of limitations, that stand in the way of its widespread adoption. Some of these limitations stem from the mathematical apparatus used to measure the pace of aging (Galkin et al., 2020a). Some of them are inherent to the information a certain data type can possibly convey about the state of the whole organism. Another set of limitations has its root in the training set domain.

Currently, all aging clocks are implemented within the supervised learning framework. A data set is collected and each sample is labeled with a target variable representing biological age. The most commonly used target variable is chronological age (CA), however more complex target variables have also been tried with great success (Levine et al., 2018; Lu et al., 2019).

A loss function is used to iteratively train a statistical model to correctly predict the age of a person until the best parameters for the model are established. When CA is used to train the model, the pace of aging is expressed as its prediction error: if an aging clock estimates someone to be five years older than their CA, their pace of aging is said to be increased.

All samples within a training set need to share a degree of similarity (Chicco, 2017). Otherwise, any modeling approach may not be able to identify the connection between the measured properties and aging.

Standard practices for putting together a training set include collecting samples from the same species, tissue, obtained with similar protocols, and using identical (or at least comparable) equipment and laboratory techniques.

In the meantime, if all samples are too similar the resulting aging clock can not be generalized (Goh et al., 2017). An aging clock trained with samples from people of the same ethnicity may not translate to another one. If all samples belong to a certain age bracket, an aging clock will likely have poor performance in people outside its range. If an aging clock is trained using only blood samples, it will not be applicable to the samples of other tissues, due to tissue-specific methylation patterns (Thompson et al., 2010).

Each choice taken to balance the similarity-diversity trade-off limits the range of possible applications of the aging clock. In this article, we display a simple approach that has let us overcome the original tissue domain of an aging clock. DeepMAge is a deep-learning DNA methylation (DNAm) aging clock, which was trained exclusively on human blood DNAm profiles (Galkin et al., 2020b). For such profiles, DeepMAge can predict CA within a 2.77 years error margin. But in saliva samples, its performance drops drastically.

However, using saliva samples for epigenetic analysis is more attractive than blood due to the simplicity of collecting the material. Saliva is also a good source of high-quality DNA for use in (epi)genomic studies and contains a broad range of diagnostically relevant molecules, such as microRNA, RNA antibodies, and inflammations markers (Langie et al., 2017).

Saliva also contains both white blood cells and buccal cells and can be considered a multi-tissue sample. Some clocks, originally trained in multiple tissues, can handle both these cell types, and thus, are suitable for epigenetic research in saliva (Fitzgerald et al., 2021). In the meantime, single-tissue models may be unable to handle the variable cell composition of saliva.

The immune cell fraction in saliva samples depends on the level of inflammation, donor’s age, and sample collection procedure (Aps et al., 2002; Theda et al., 2018). The effect of cell composition may obscure the useful information contained within epigenetic profiles. There are multiple cell-type deconvolution tools that allow to diminish this effect (Langie et al., 2017; Houseman et al., 2012). We used one such reference-based tool—EpiDISH—to derive a linear cell-type adjustment to DeepMAge’s predictions (Teschendorff et al., 2017). With this adjustment, the aging clock’s accuracy was salvaged, showing that in some cases it is not necessary to include different tissues into the training set of a deep-learning model to obtain a multi-tissue aging clock.

## 2 Materials and Methods

### 2.1 Aging Clock

The aging clock used in this study is DeepMAge originally published in Galkin et al. (2020b). DeepMAge is a deep-learning neural network that takes in a vector of *β*-values for 1,000 CpG sites present in Illumina BeadChip 27 K and Illumina BeadChip 450 K platforms.

DeepMAge has been trained on a collection of blood DNAm profiles and its behavior in saliva samples has not been described elsewhere.

### 2.2 Cell Type Deconvolution

To determine the cell type composition of the saliva samples, EpiDISH described in Zheng et al. (2018) was used. EpiDISH is available as an R package at https://github.com/sjczheng/EpiDISH (v.2.6.0).

### 2.3 Data Collection

All data used in this study is publicly available at Gene Expression Omnibus (GEO). We selected the datasets according to the following criteria: 1) A data set had to contain epigenetic profiles obtained with an Illumina Infinium array; 2) A data set had to contain saliva and/or buccal swab samples; 3) A data set had to be annotated with age and sex information.

In the end, 12 datasets were selected. Study accession numbers for the training set are: GSE78874–contains men and women of two ethnic backgrounds: Hispanics and Caucasians (*N* = 259 people), GSE80261–contains human buccal epithelial cells from children from the NeuroDevNet cohort (*N* = 216), GSE94876–contain methylation profiles in buccal cells of long-term smokers and moist snuff consumers (*N* = 120), GSE34035–contains saliva DNA from alcohol-dependent subjects (*N* = 112), GSE50759–contains samples from buccal epithelium collected using exfoliative brushing (*N* = 96), GSE28746–saliva samples from male identical twins discordant for sexual orientation (*N* = 84), GSE42700 - buccal cell samples collected at birth and 18 months from 10 monozygotic and five dizygotic twin pairs from the Peri/postnatal Epigenetic Twins Study (PETS) cohort (*N* = 53), GSE50586–buccal swabs (*N* = 20). Studies used in the verification set include: GSE92767–saliva from 54 males aged 18–73 years (*N* = 54), GSE109042–human buccal epithelial cells from children with fetal alcohol spectrum disorder and control samples (*N* = 47), GSE28217–contains primary oral squamous cell carcinoma, oral leukoplakia, and normal oral mucosa (*N* = 12). GSE48472–contains data from blood, saliva, buccal swab, and hair follicles, but only saliva and buccal swab samples were used (*N* = 5). All samples were normalized using lumi, according to the protocol described in Galkin et al. (2020b), Du et al. (2008).

### 2.4 DeepMAge Prediction Adjustment

Several approaches were compared to find the optimal way of enabling accurate DeepMAge predictions for saliva samples:

• **No adjustment**: DeepMAge predictions were used as-is;
• **Simple adjustment**: No information from cell-type deconvolution was used. Actual age of the saliva samples was regressed as a function of its DeepMAge prediction alone;
• **Shift adjustment**: Delta_Age_ was regressed as a function of saliva samples’ cell types to obtain a correction term, where Delta_Age_ = DeepMAge prediction–CA. Correction terms were added to DeepMAge output of the saliva samples to obtain the adjusted predictions;
• **Total age adjustment**: CA of saliva samples was regressed as a function of DeepMAge predictions and cell composition to obtain the adjusted predictions.

Any models used to derive adjustments were implemented with the ElasticNet fitter, as implemented in scikit-learn v.0.24.1 Python package. Optimal regression parameters were chosen based on the grid search defined as:

{′l1_ratio′: numpy.arange(0, 1, 0.01),

′alpha′: (1e-5, 1e-4, 1e-3, 1e-2, 1e-1, 0.0, 1.0, 10.0, 100.0)}

with leave-one-study-out (LOSO) cross-validation (CV) folds. The final adjustment regressions were trained on all studies, save those in the verification set.

For simple and total age adjustments, transformed predicted age values were tried. The adjustment was described in detail in Horvath (2013). The age transformation may be expressed as:

$$f\left(x\right)\;=\left\{\begin{array}{c} \frac{x+1}{21}-1,\;if\;x>20\;\\ \ln\frac{x+1}{21},\;if\;x\leq 20\; \end{array}\right.$$

$$f^{-1}\left(x\right)\;=\left\{\begin{array}{c} 21\times exp\left(x\right)-1,\;if\;x<0\;\\ 21\times x+20,\;if\;x\geq 0\; \end{array}\right.$$

## 3 Results

### 3.1 Cell Composition in Saliva DNA Methylation Studies

**FIGURE 1:**
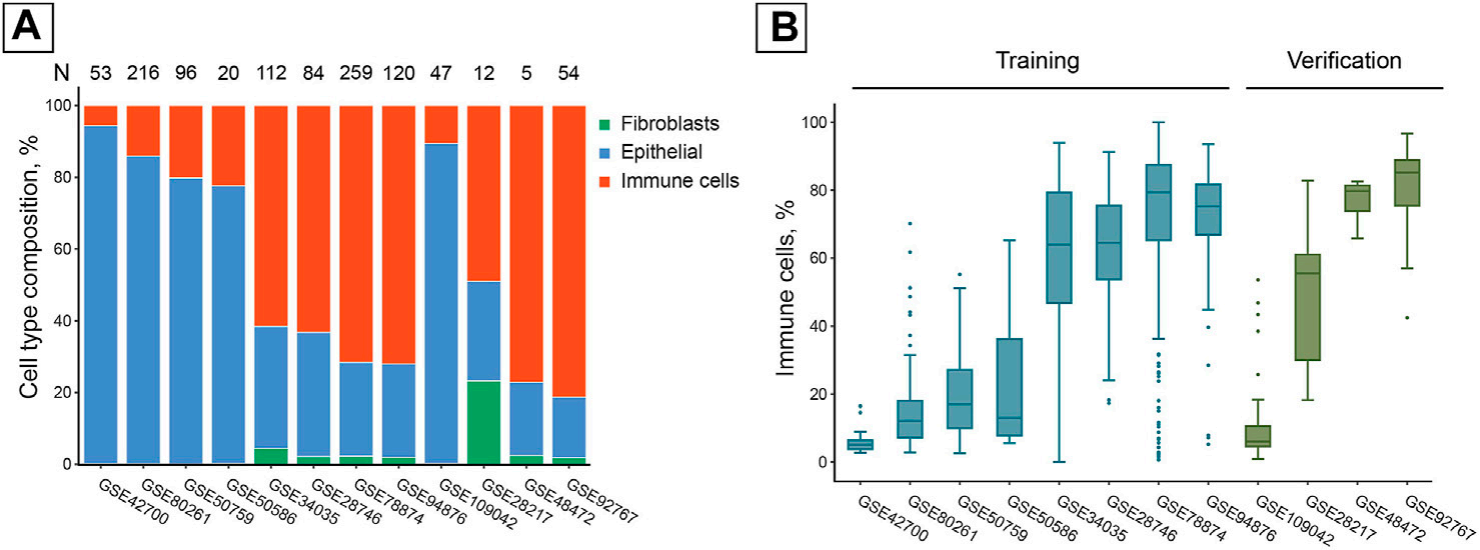
Publicly available studies of DNAm in saliva samples have high inter- and intra-study cell composition variability. **(A)** Average cell composition of saliva samples in the studies used, estimated with EpiDISH DNAm deconvolution. N = Total number of samples in a study. **(B)** Immune cell distributions within the studies used. Boxes correspond to the IQR, whiskers protrude no further than 1.5×IQR. Boxes are colored according to their inclusion in the training or verification set.

The collection of publicly available studies we used was extremely diverse in its cell composition. The average fraction of immune cells in the samples varied within the 5.6–81.3% range. Most studies contained a negligible fraction of fibroblasts (< 5%), except for GSE28217 in which samples contained 23.3% fibroblast cells on average (Figure 1A).

Cell composition varied significantly not only between studies but also between the samples from the same study, illustrating the heterogeneity inherent to salivary samples processed according to the same study protocol (Figure 1B).

### 3.2 Enabling Age Prediction in Saliva Samples

No adjustment for cell-type composition produced worse predictions than the baseline model – median age assignment (Table 1). The accuracy was increased with any of the proposed adjustments. Among the adjusters using non-transformed age, the correction based on delta age, immune and epithelial cell relative counts (“Delta age” in Table 1) produced the most accurate results – MAE = 6.34 years in the CV and MAE = 6.28 years in the verification sets.

**TABLE 1:** All cell-type variability adjustments significantly improve DeepMAge’s accuracy in saliva samples MAE = Mean Absolute Error, see MATERIALS AND METHODS for the description of the adjustments used.

Adjustment	Age transformed	MAE, years	
		Train	Test
Baseline	Non-transformed	22.09	19.03
None	Non-transformed	26.36	20.86
Simple	Non-transformed	13.48	13.74
	Transformed	12.54	13.87
Delta age	Non-transformed	6.34	6.28
Total age	Non-transformed	7.76	6.83
	Transformed	4.57	4.74

**FIGURE 2:**
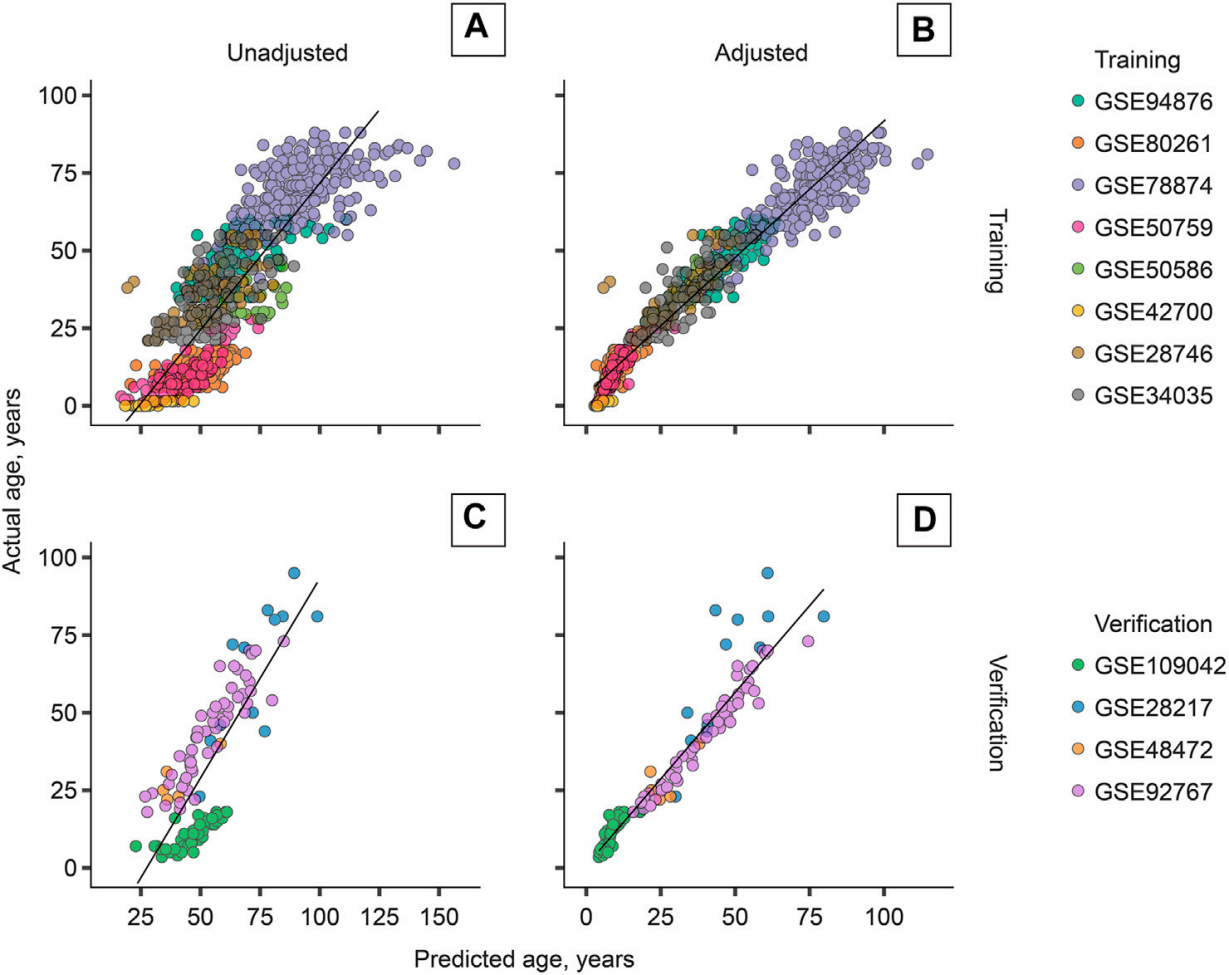
Cell-type adjustment (“total age-transformed”) significantly improves the performance of DeepMAge (originally developed for blood samples) in the domain of saliva samples. **(A)** Training set, unadjusted: *R* ^2^ = 0.73. **(B)** Training set, adjusted: *R* ^2^ = 0.95. **(C)** Verification set, unadjusted: *R* ^2^ = 0.63. **(D)** Verification set, adjusted: *R* ^2^ = 0.92. *R* ^2^ = coefficient of determination. Predictions for the training set were obtained with leave-one-study-out cross-validation.

**FIGURE 3:**
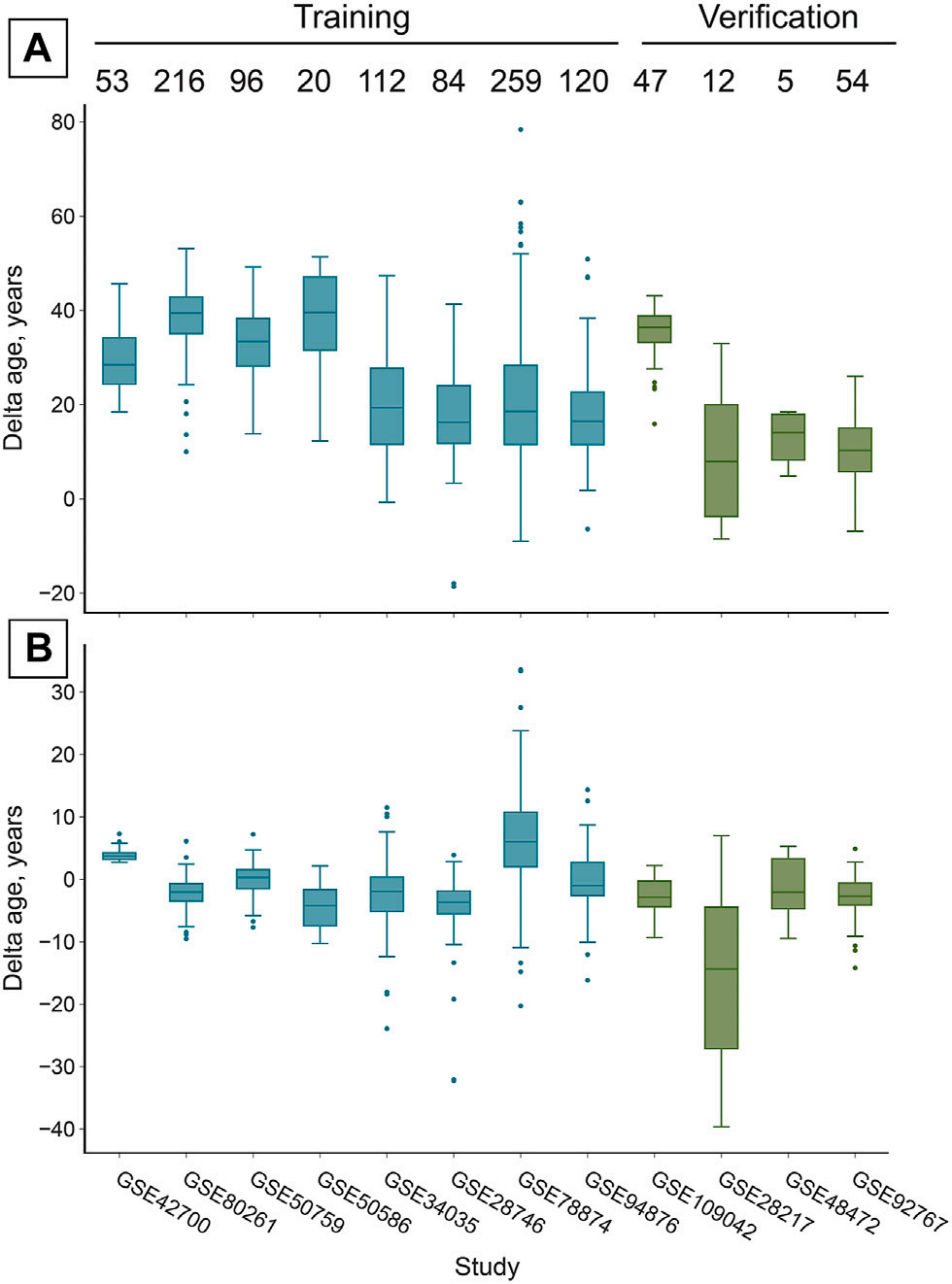
The reported adjustment significantly increases the accuracy of age prediction in all studies, despite their different cell-type composition (Figure 1). **(A)** Prior to the adjustment *DeepMAge*_*Blood*_ predicts chronological age in saliva samples with a MAE of 26.36 years in the training set and 20.86 years in the verification set (Table 1). Numbers above boxes stand for the number of samples in each study. **(B)** After the “total age-transformed” adjustment DeepMAge’s error in saliva samples drops to a MAE of 4.57 years in the training set and 4.74 years in the verification set, despite the variable cell type composition of the samples across studies (Figure 1A). Boxes correspond to the interquartile region (IQR), whiskers protrude no further than 1.5×IQR. Boxes are colored according to their inclusion in the training or verification set.

The most accurate adjuster, however, used the age transformation described in Methods. The best adjuster among those tested is “Total age-transformed” with a MAE of 4.57 years in the CV set and 4.74 years in the verification sets (Figure 2). This adjustment improved DeepMAge’s accuracy in all tried data sets, despite their differences in cell-type composition (Figure 3).

The total age adjustment can be expressed as the following formulas, with and without age transformation, respectively:

$$f\left(DeepMAge_{\;Saliva}\right)=0.93\times f\left(DeepMAge_{\;Blood}\right)-1.14\times Epi+1.05\times IC-1.18$$ (1)

$$DeepMAge_{\;Saliva}=0.85\times DeepMAge_{\;Blood}-11.54\times Epi+10.56\times IC-15.76$$ (2)

where Epi and IC are the portions of epithelial and immune cells in the sample, respectively.

### 3.3 The Effect of Immune Cell Fraction on the Increase in Accuracy

Since DeepMAge was trained on a set of blood-derived DNAm profiles, we wondered if our adjuster-suggested shift in prediction was larger in samples with lower IC proportions. For this purpose, we measured the increase in accuracy between *DeepMAge*_*Blood*_ and *DeepMAge*_*Saliva*_ as the drop in distance to the chronological age after the adjustment.

**FIGURE 4:**
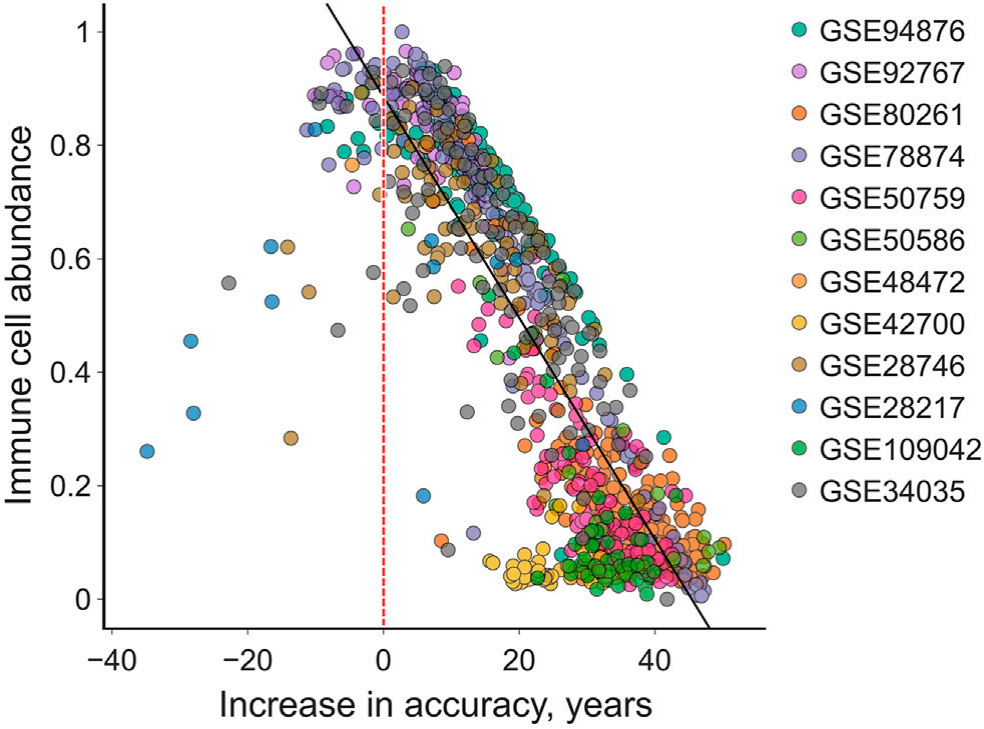
The “total age-transformed” adjustment increases the age prediction accuracy in individual samples by up to 50 years in saliva samples. The absolute increase in accuracy is smaller in samples with higher immune cell count. Very few (5%) samples are predicted less accurately (left to the red vertical line) after the adjustment. Black line is the ordinary least squares regression, its *R* ^2^ is 0.72 and Pearson’s r is −0.85.

We compared the increase in accuracy to the IC content of the samples to discover that IC content was strongly negatively correlated with the absolute adjustment magnitude (*R* ^2^ = 0.72, *Pearson*′*s r* = −0.85). Moreover, only 5% of all samples actually grew farther from the chronological age, among which most consist of > 50% IC (Figure 4).

## 4 Discussion

In this article, we have demonstrated a method to expand an aging clock’s domain of applications to other tissues.

More specifically, the method we propose can be used to translate blood-trained aging clocks to saliva and buccal swab samples.

Saliva is one of the easiest to obtain tissues and is a good source of high-quality DNA. In comparison to blood, saliva requires no additional anticoagulant, the risk of disease transmission is lower, and there is no need for venipuncture (Langie et al., 2017). Although blood is the most widely used tissue for clinical tests, saliva in its own right has been shown to be a valuable biomarker for the early detection of such cancer types as oral cancer, head and neck squamous cell carcinoma, and breast cancer (Liyanage et al., 2019; Bryan et al., 2013; Nagler, 2009; Delmonico et al., 2015).

Moreover, saliva methylome was also shown to be positively correlated with methylation in blood for 88.5% (Smith et al., 2015). Thus, changes in salivary DNAm profiles might be indicative of organism-wide aging processes that can also be detected in blood DNAm levels. From a technical standpoint, however, saliva cannot be used as a substitute for DNAm aging clocks trained on blood samples only.

DeepMAge is a deep learning model, which was trained on a set of blood-derived DNAm profiles and shows poor performance in saliva samples. Salvaging its accuracy in other tissues can be formalized as a domain adaptation problem, for which a variety of theoretical solutions exist within the deep learning framework. Our solution, however, does not involve retraining the model or manipulating the target domain samples. It is based on introducing additional information (cell type composition) on the target domain samples to derive a linear offset for aging clock predictions. The reported adjustment has resulted in a saliva DNAm aging clock with a MAE of 4.7 years, which is comparable to the clock described in Bocklandt et al. (2011) which was trained in saliva and reached a MAE of 5.2 years.

To estimate the cell content of salivary sample, we used EpiDISH – a cell type deconvolution algorithm that uses a reference of pure cell type DNAm profiles to evaluate the cell composition of bulk samples. This tool was verified using *in silico* mixtures of pure cell types. Its creators aimed to remove the effect of immune cell contamination in differential methylation studies in epithelial tissues.

Since DeepMAge was trained on blood data, the IC effect represents not the contamination, in this setting, but the signal the aging clock is prepared to recognize. Thus in some sense, EpiDISH may be said to adjust for the noise associated with epithelial DNAm profiles, bringing the samples closer to the original training domain.

However, this explanation for the efficiency of cell-type adjustment in the context of DeepMAge performance is an oversimplification. EpiDISH does not deconvolute DNAm bulk profiles into three distinct profiles of fibroblast, immune, and epithelial origin, the mixture of which would produce the observed profile. It only provides a vector of relative abundances for these three cell types in the sample. Thus, it is impossible to subtract the epithelial noise from the DNAm profiles. Another counterargument for the noise hypothesis is that EpiDISH significantly improves DeepMAge prediction quality even in data sets that have low immune cell abundance (GSE42700, GSE109042), and thus could be said to have an overwhelming noise to signal ratio (Figure 4).

Another, more probable explanation for the efficiency of EpiDISH is that the pace of epigenetic aging, as perceived by DeepMAge, is faster in epithelial samples. This follows from the total age correction formula: 1) DeepMAge overestimates the age of saliva samples (see <1 coefficient for DeepMAge _Blood_, negative intercept), 2) a sample consisting completely of epithelial cells would be predicted 27.3 years older than it should be (see negative *Epi* coefficient, negative intercept).

Another major contributing factor, apparently, is that despite their different function and ontogenesis, buccal epithelium and immune blood cells share similar aging signatures.

Since the correction can be interpreted to stretch the original DeepMAge prediction space, it may be argued that the changes in the organismal pace of aging may be more rapidly reflected in saliva than in blood. This quicker responsiveness and the difference in the aging rate between IC and epithelium may be explained by a much higher turnover rate of buccal epithelium whose surface layer is replaced every 3 h (Sender and Milo, 2021; Dawes, 2003).

The tissue-specific pace of aging has been reported in some other publications (Hannum et al., 2013; Thompson et al., 2018), yet in them, the impact of variable cell composition is not explored. We believe that it is important to delineate the effects of the primary tissue and that of the contaminating cells.

We tried the approach similar to the one used in Hannum et al. (2013) (“Simple adjustment” in Table 1) (two-fold increase in accuracy). While it yielded better results than the non-adjusted DeepMAge, the best adjuster produced predictions almost three times as accurate (Table 1). We argue that simple tissue-specific linear offsets are insufficient to turn a single-tissue clock into a multi-tissue one. This approach ignores the marked variance of cell composition that is present even within the same specimen collection protocol. Samples from the same study can have no immune cell contaminants at all, or be composed of them completely (Figure 1). Nonetheless, both extremes and everything in-between will be identically labeled as “saliva”.

In the future, single-cell DNAm profiling may render the concept of a “tissue-specific” aging clock obsolete by designing biological age as a probabilistic function of individual cells (Trapp et al., 2021). But within the bulk sample context, DNAm deconvolution is an impressively efficient technique to solve the tissue domain problem. While working with public data repositories, it can also serve as a quality control tool to remove outliers, control a hidden source of technical variation, and fill in missing or misleading tissue labels.

From a practical standpoint, our results show that it is not necessary to retrain deep learning clocks or employ any other complex domain adaptation techniques to widen their range of use cases. Since the correction procedure is model agnostic, similar extensions may be developed for shallow learning aging clocks.

While blood is easily available in clinical settings, the necessity to schedule blood drawing stands in the way of consumer biogerontology applications. Using cell deconvolution to adapt the existing blood domain solutions to saliva samples will increase the adoption rate of the aging clock technology.

## 5 Conclusion

Cell-type deconvolution can be used to expand the applicability of aging clocks to the tissues outside of their original training domain and significantly increase their accuracy for initially unintended use-cases.

## Data Availability

The datasets presented in this study can be found in the Gene Expression Omnibus online (GEO) repository. The accession numbers can be found below: GSE78874, GSE80261, GSE94876, GSE34035, GSE50759, GSE28746, GSE42700, GSE50586, GSE92767, GSE109042, GSE28217, GSE48472.
